# Supplementary material for: Antimycobacterial and Nitric Oxide Production Inhibitory Activities of Triterpenes and Alkaloids from Psychotria nuda (Cham. & Schltdl.) Wawra
Source: Molecules. 2019 Mar 15;24(6):1026. doi: 10.3390/molecules24061026 (PMC6471101; doi:10.3390/molecules24061026)
Supplement: Supplementary file 1 [file molecules-24-01026-s001.pdf]

Supplemental Materials

Antimycobacterial and Nitric Oxide Production Inhibitory Activities of Triterpenes and Alkaloids from *Psychotria nuda* (Cham. & Schltdl.) Wawra

Almir R. de Carvalho Juniora[1*], Rafaela O. Ferreira[2], Michel de S. Passos[3], Samyra I. Boeno[3], Lorena L. Glória das Virgens[3], Thatiana L. Biá Ventura[4], Sanderson Dias Calixto[4], Elena Lassounskaia[4], Mario Geraldo de Carvalho[5], Raimundo Braz-Filho[3,5], Ivo J. Curcino Vieira[3]

[1]Instituto Federal de Santa Catarina, Câmpus Criciúma, Criciúma, SC, Brazil. almir.ribeiro@ifsc.edu.br.
[2] Colegiado de Ciências Exatas e Biotecnológicas, Universidade Federal do Tocantins, Gurupi, TO, Brazil
[3] Laboratório de Ciências Químicas, Centro de Ciência e Tecnologia, Universidade Estadual do Norte Fluminense Darcy Ribeiro, Campos dos Goytacazes, RJ, Brazil
[4] Laboratório de Biologia do Reconhecer, Universidade Estadual do Norte Fluminense Darcy Ribeiro, Campos dos Goytacazes, 28013-602, Rio de Janeiro, Brazil
[5] Departamento de Química, Instituto de Ciências Exatas, Universidade Federal Rural do Rio de Janeiro, Seropédica, BrazilAffiliation 1; e-mail@e-mail.com

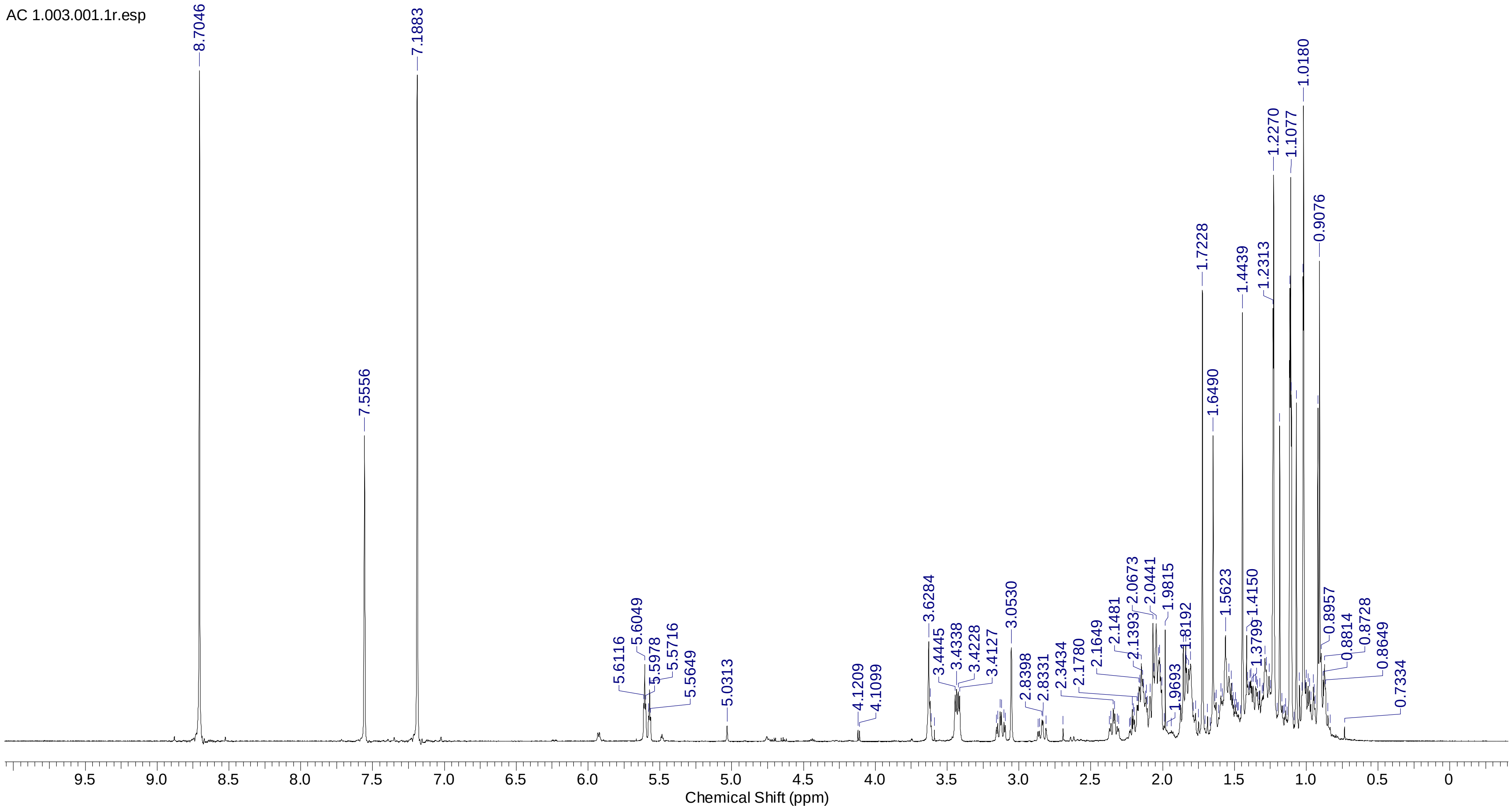

Figure S1. ^{1}H NMR spectrum (500 MHz, Pyridine-d_5) of compounds **1** and **2**.

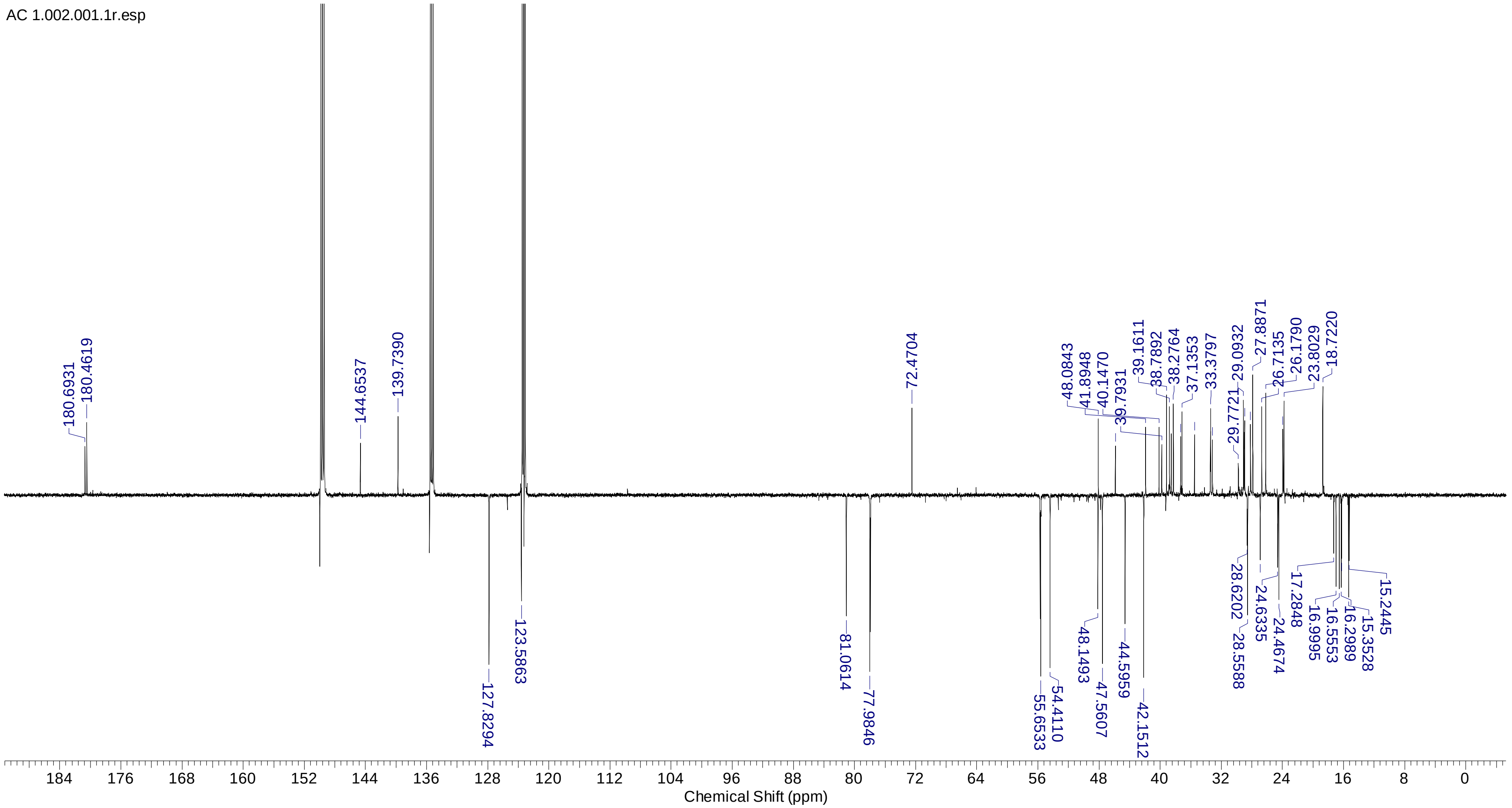

Figure S2. DEPTQ spectrum (125 MHz, Pyridine-d5) of compounds **1** and **2**.

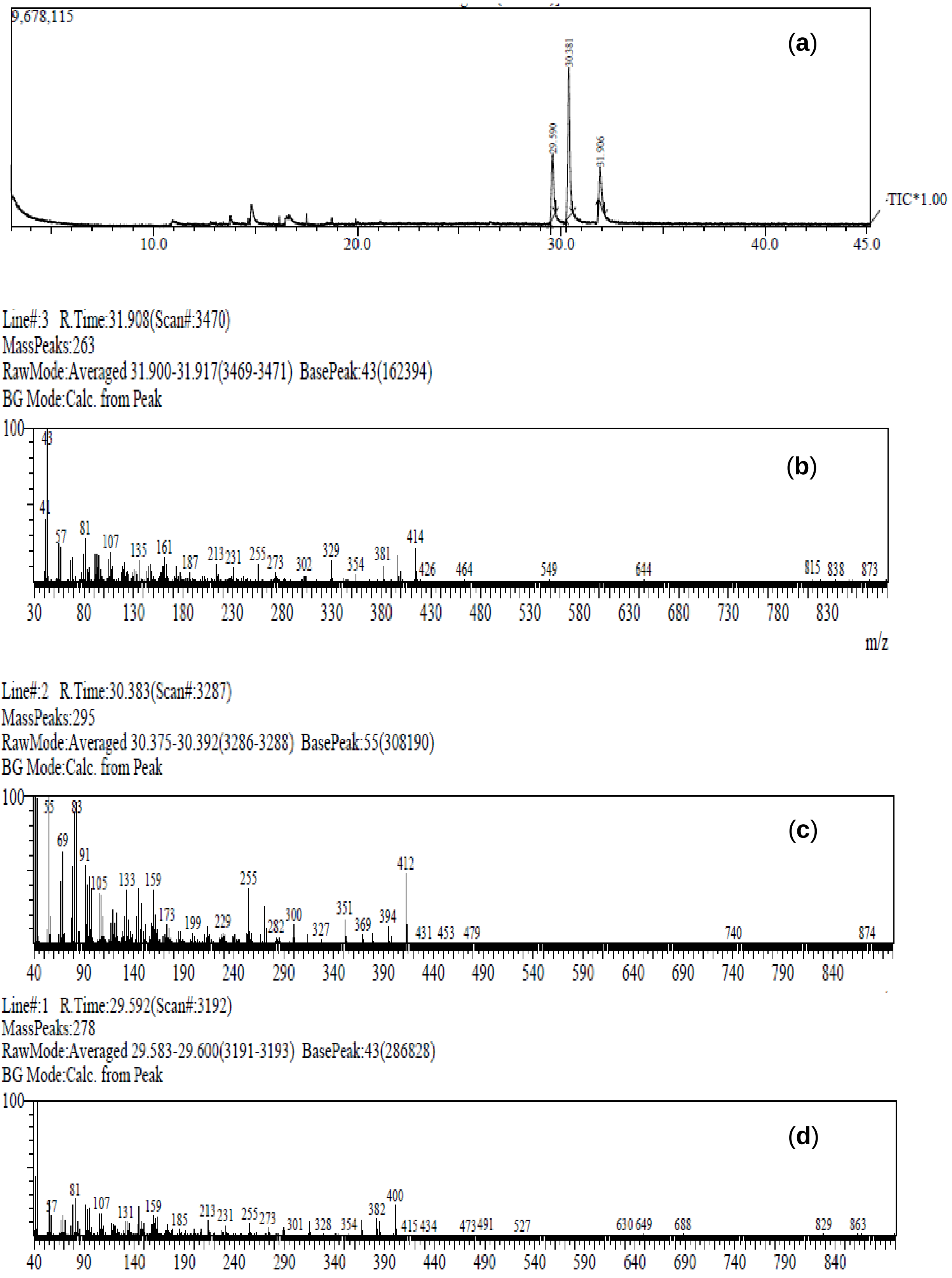

Figure S3. GC/MS chromatogram (**a**) and LRMS of compounds **3** (**b**), **4** (**c**), and **5** (**d**).

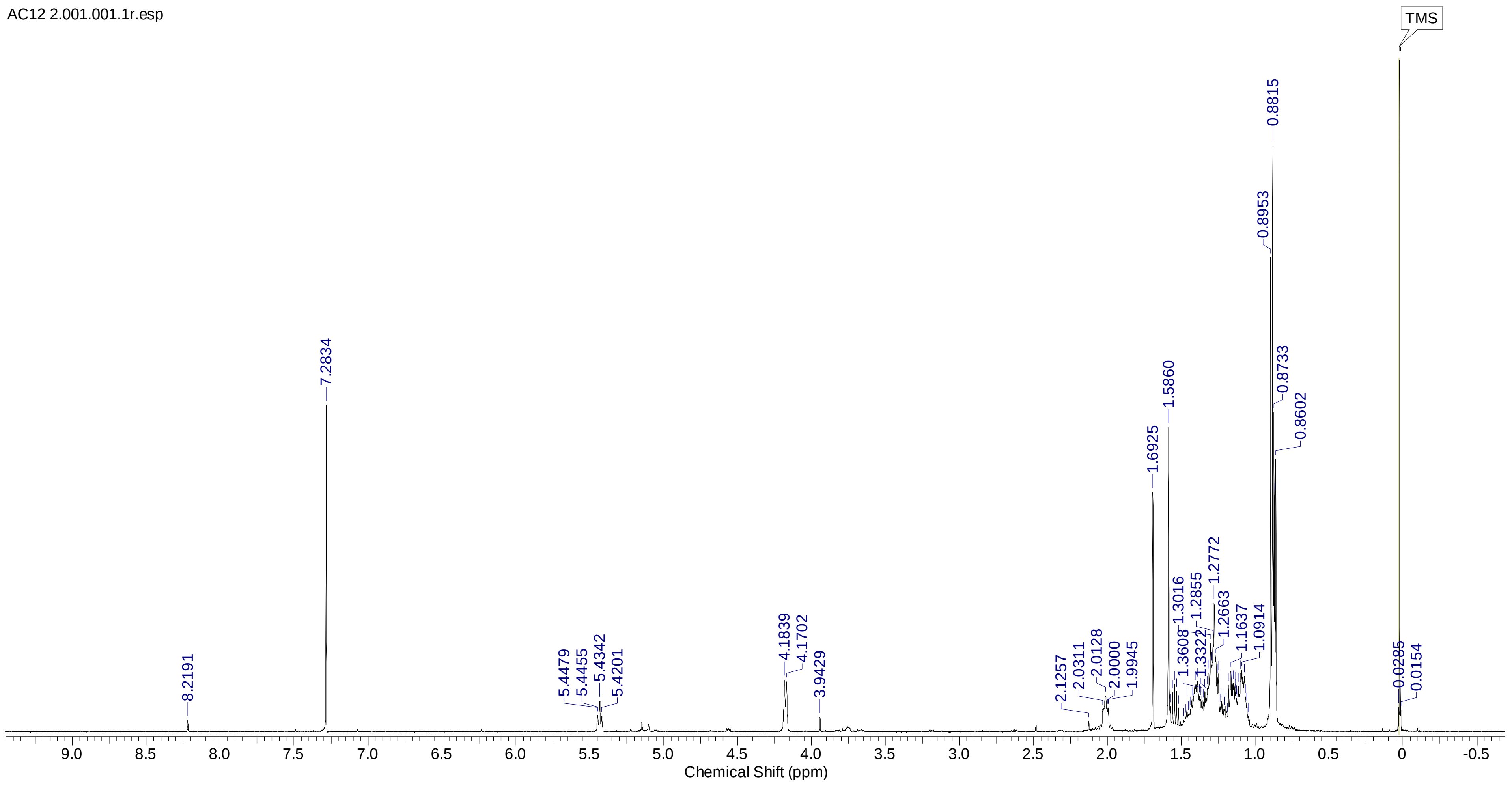

Figure S4. ^{1}H NMR spectrum (500 MHz, $CDCl_3$) of compound **6**.

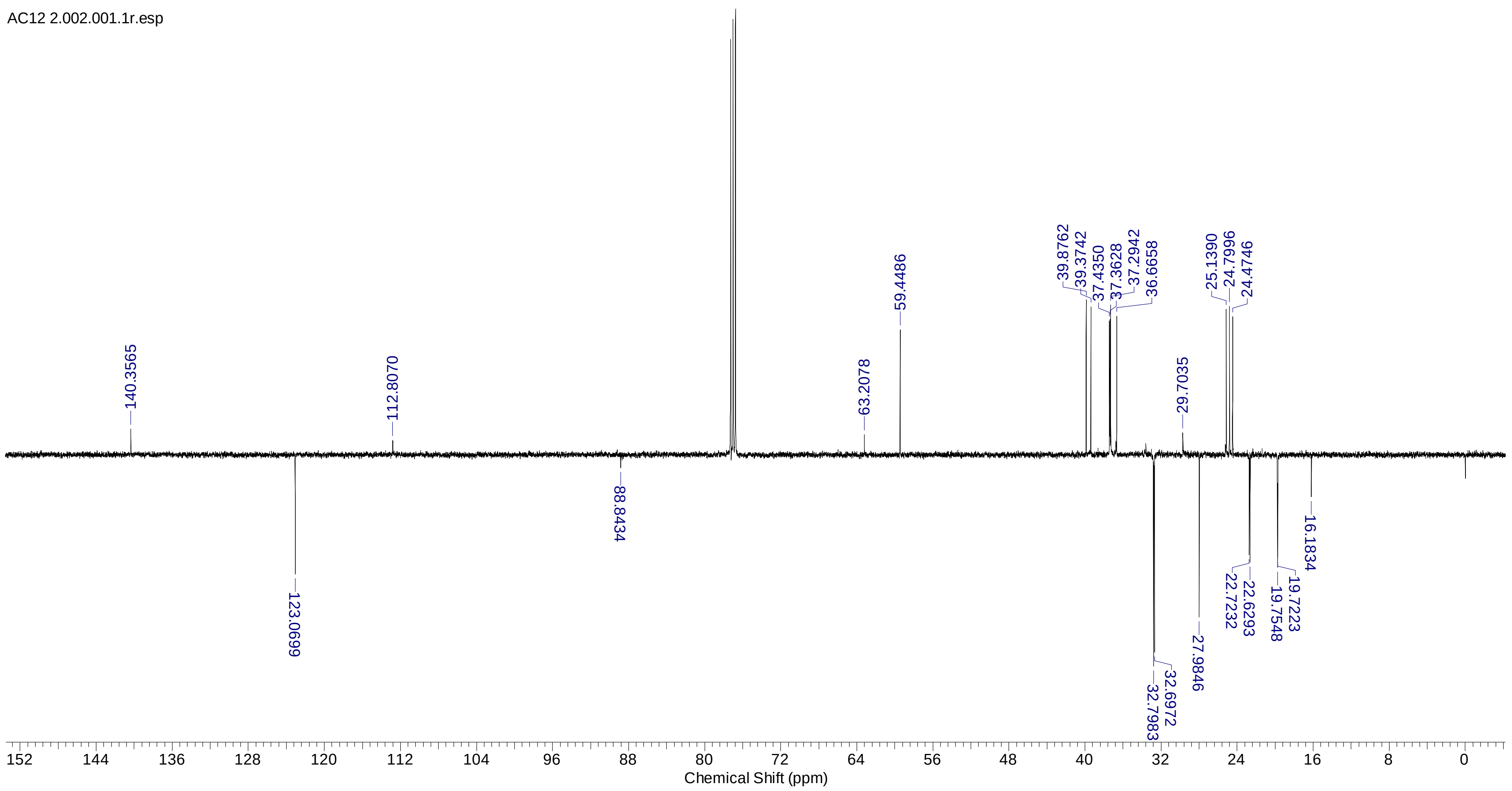

Figure S5. ^{13}C NMR spectrum (125 MHz, $CDCl_3$) of compound **6**.

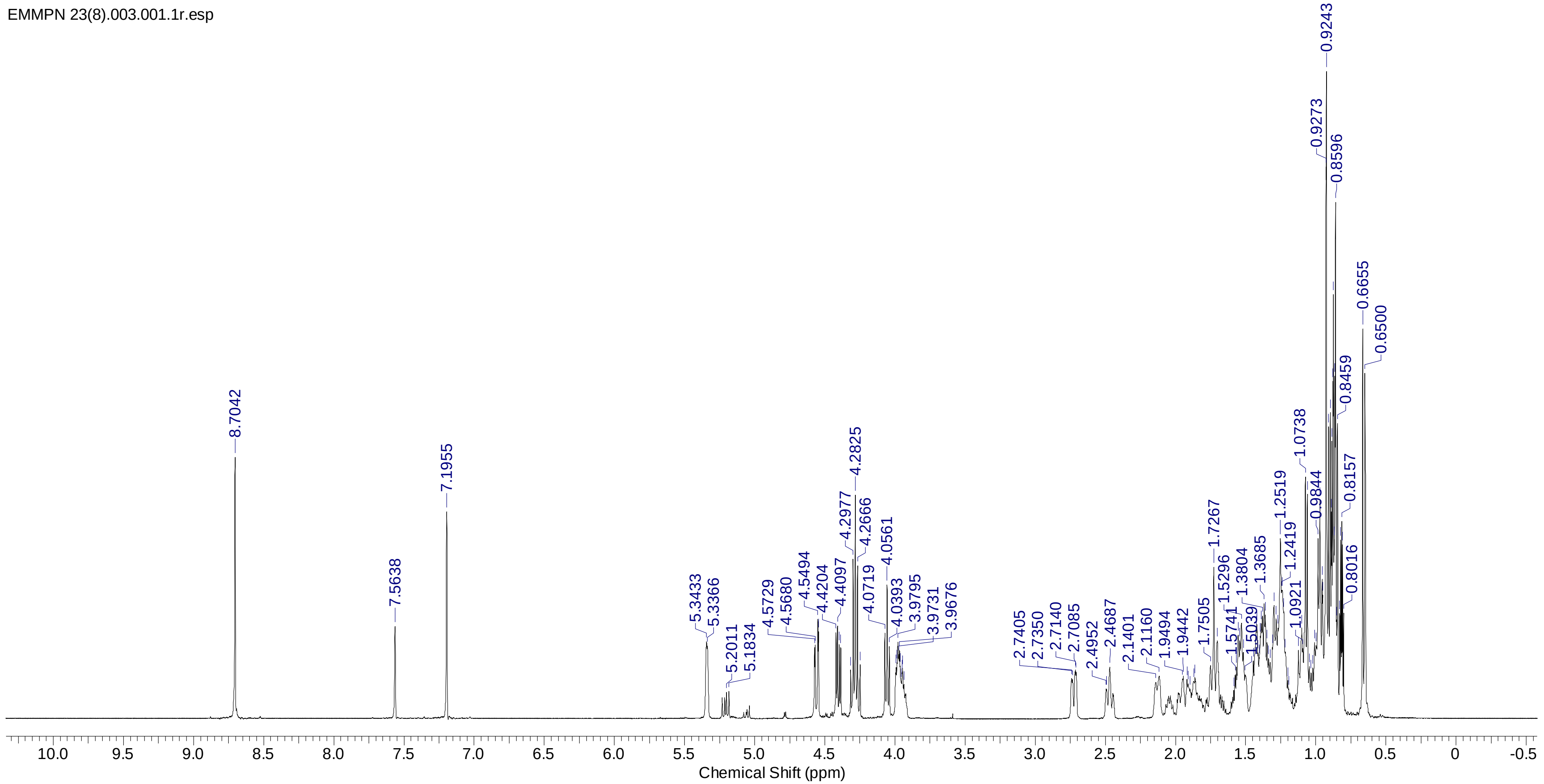

Figure S6. ^{1}H NMR spectrum (500 MHz, Py-d_5) of compounds **7** and **8**.

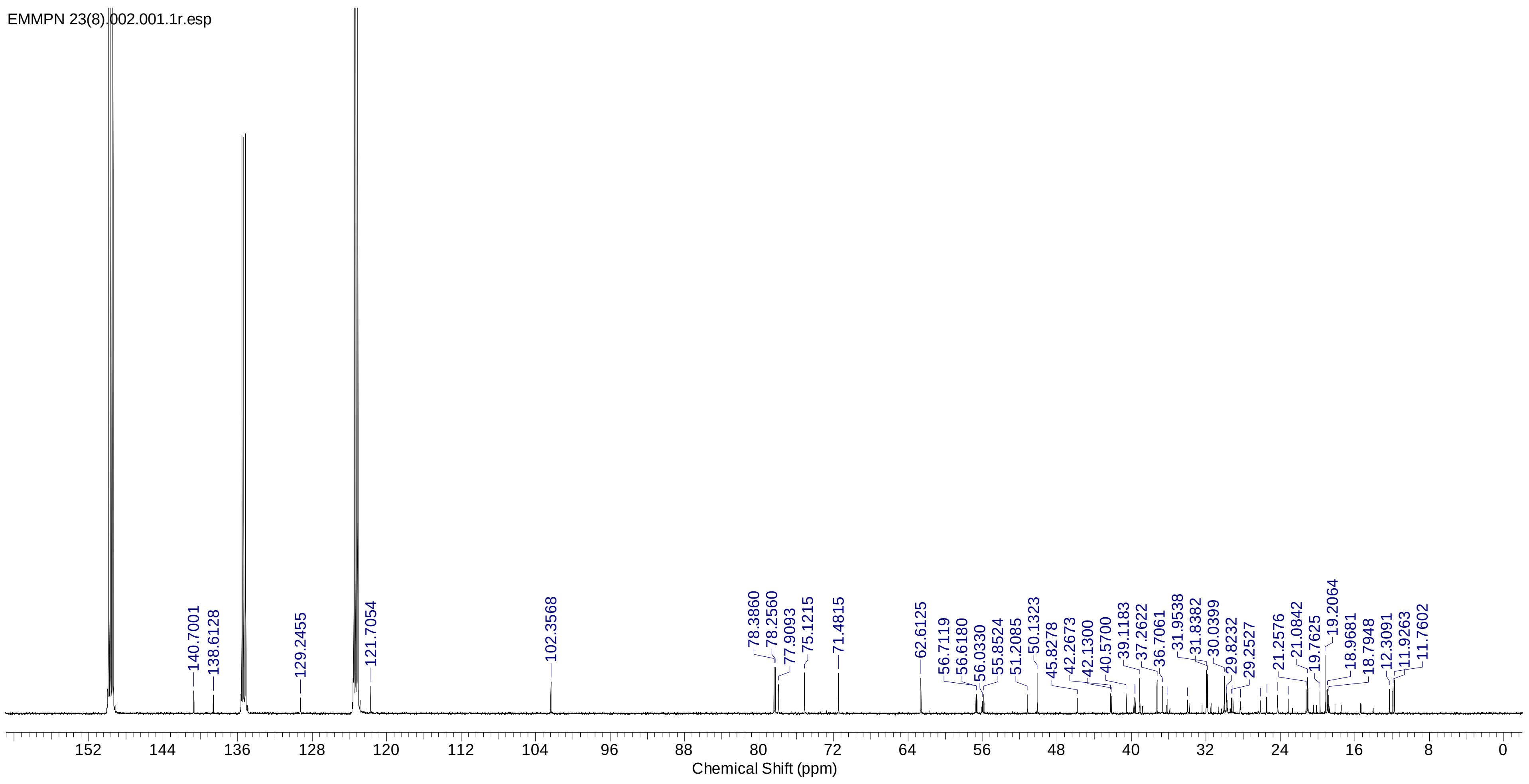

Figure S7. ^{13}C NMR spectrum (125 MHz, Py-d_5) of compounds **7** and **8**.

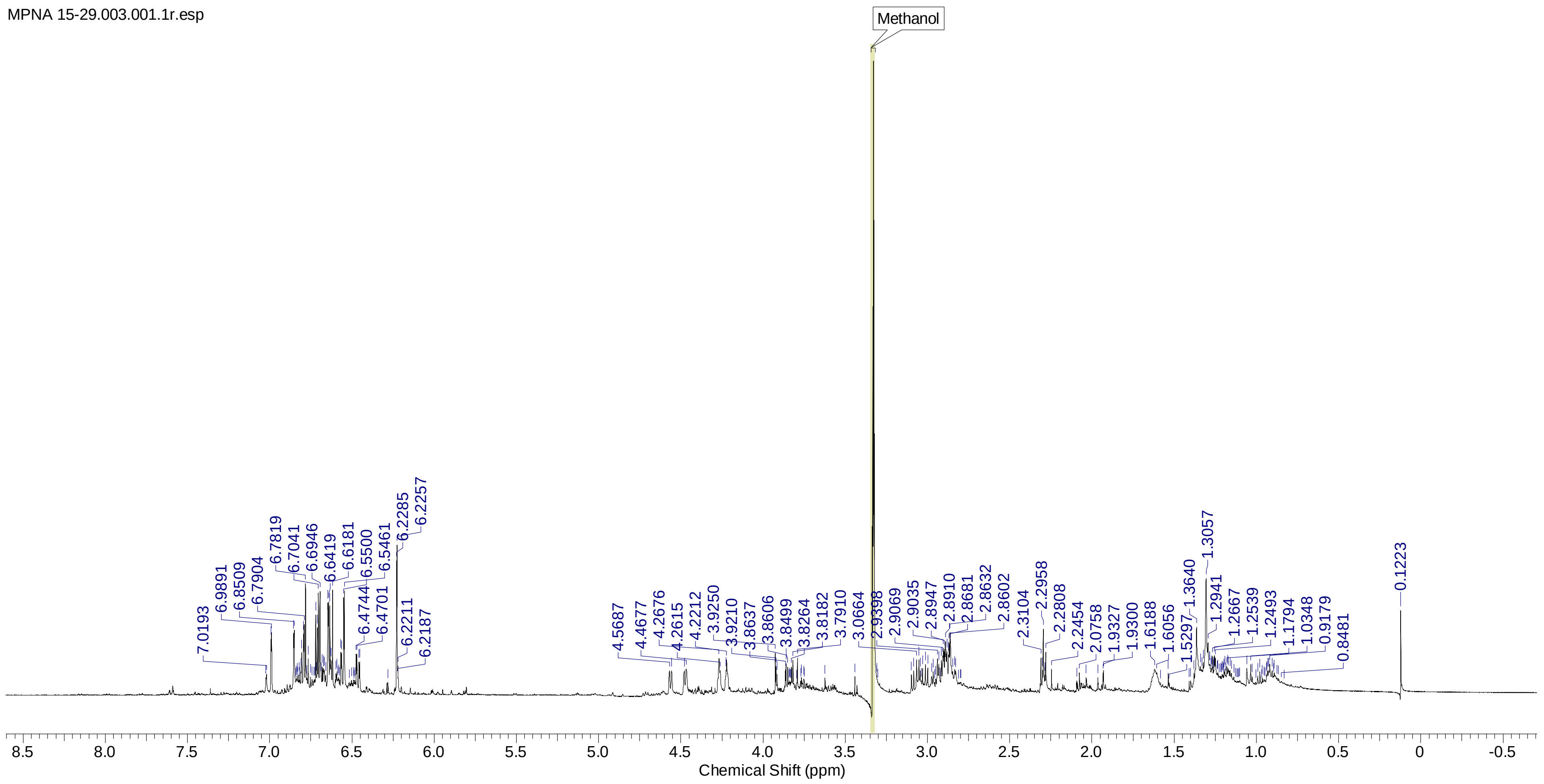

Figure S8. ^{1}H NMR spectrum (500 MHz, CD_3OD) of compounds **9** and **10**.

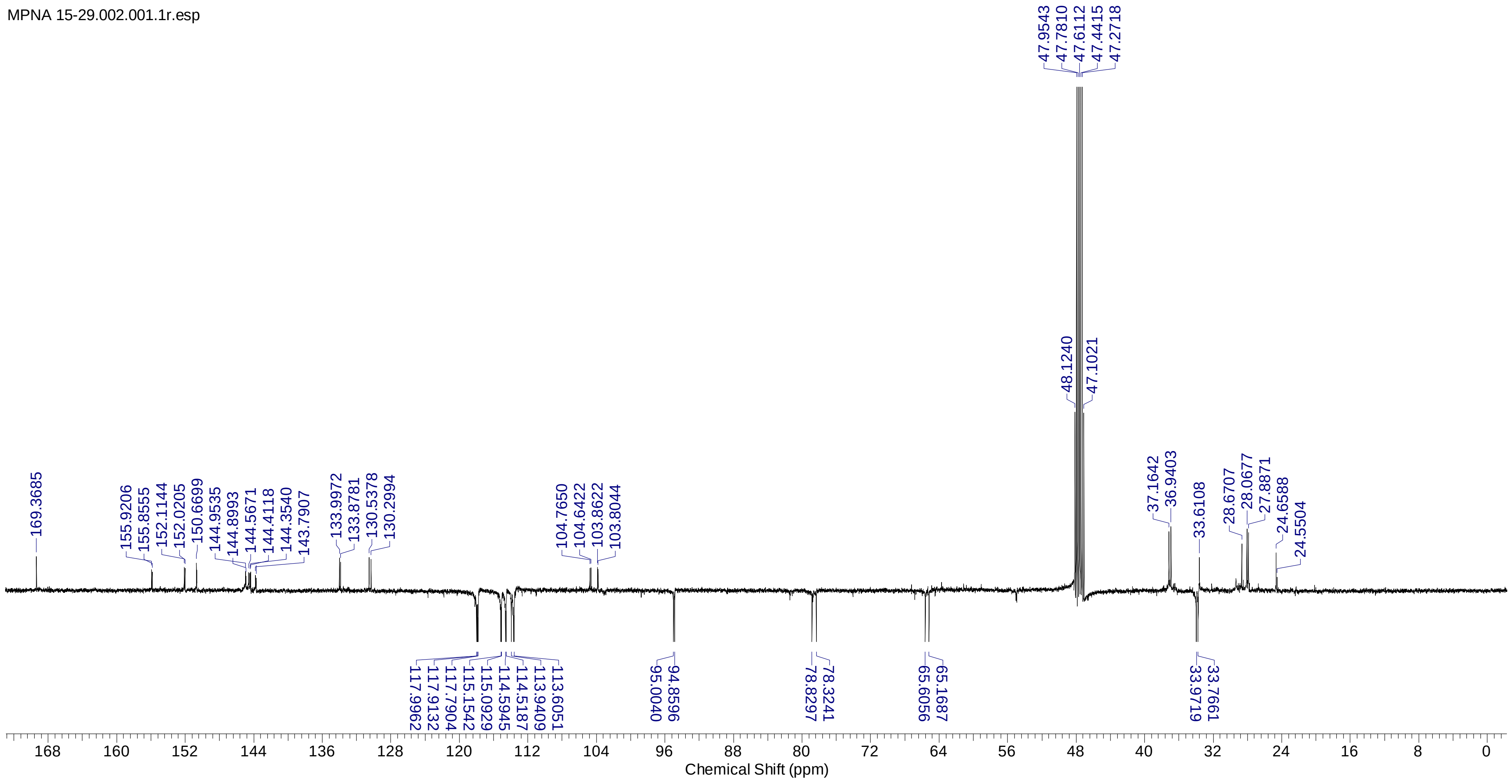

Figure S9. ^{1}H NMR spectrum (125 MHz, CD_3OD) of compounds **9** and **10**.

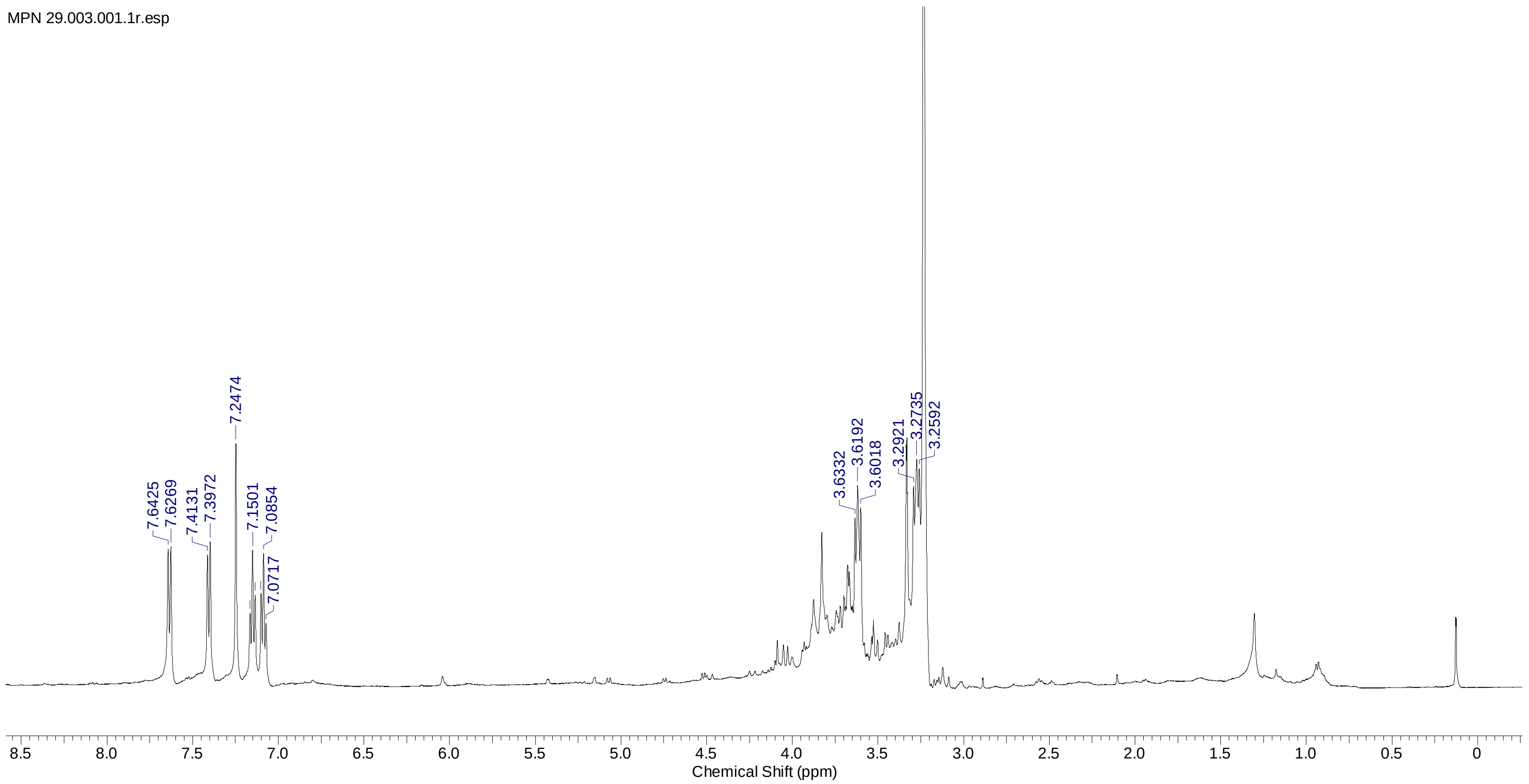

Figure S10. ^{1}H NMR spectrum (500 MHz, CD_3OD) of compound **11**.

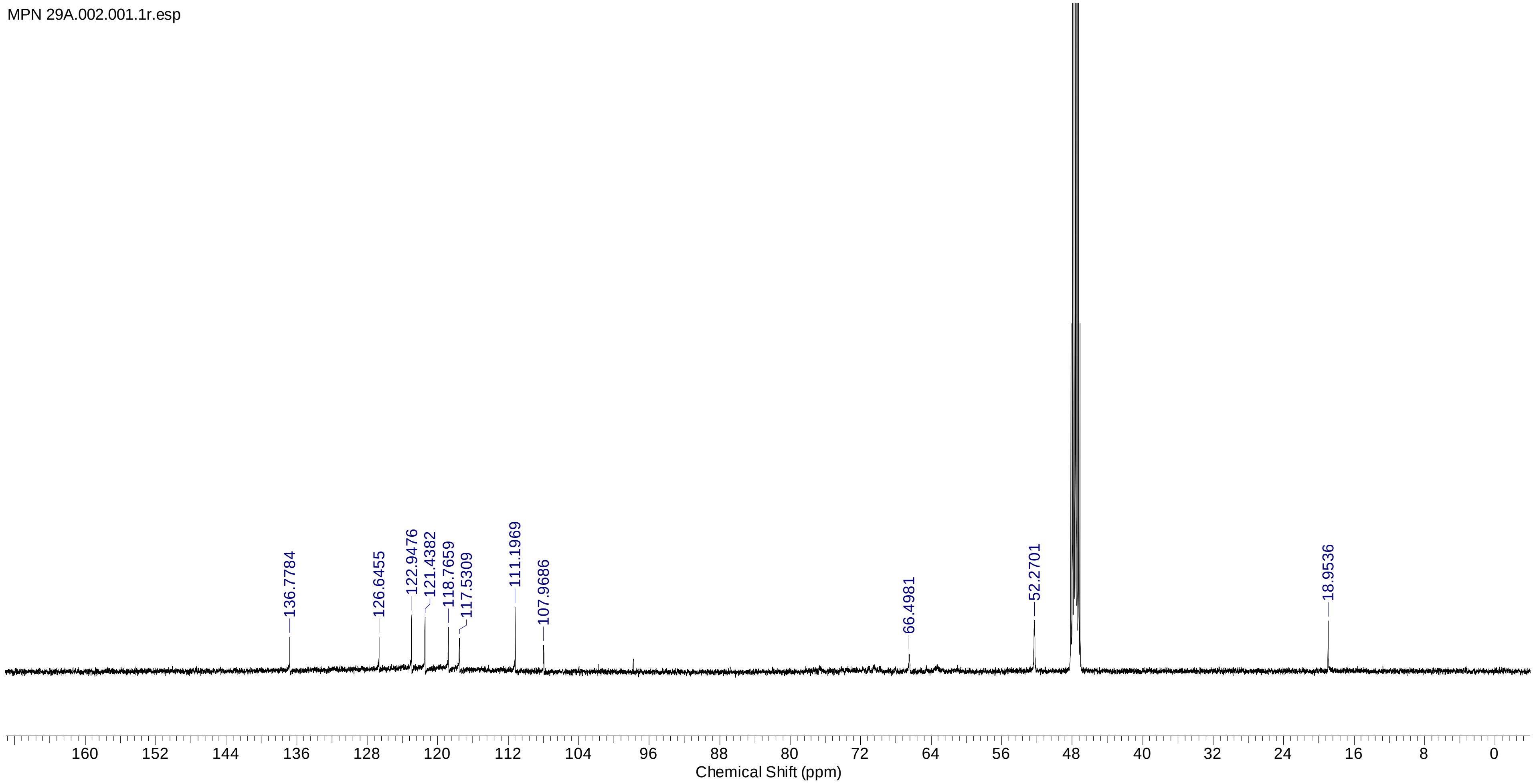

Figure S11. ^{13}C NMR spectrum (125 MHz, $CDCl_3$) of compound **11**.

Analysis Info

Analysis Name	D:\Data\Ivo Cursino\MPN29.d	Acquisition Date	9/10/2015 11:25:06
Method	tune_low_pos_LC_Chris_11_06_2014.m	Operator	UENF
Sample Name	MPN29	Instrument	micrOTOF-Q II 10374
Comment			

Acquisition Parameter

Source Type	ESI	Ion Polarity	Positive	Set Nebulizer	2.0 Bar
Focus	Not active	Set Capillary	4500 V	Set Dry Heater	200 °C
Scan Begin	50 m/z	Set End Plate Offset	-500 V	Set Dry Gas	8.0 l/min
Scan End	1200 m/z	Set Collision Cell RF	120.0 Vpp	Set Divert Valve	Waste

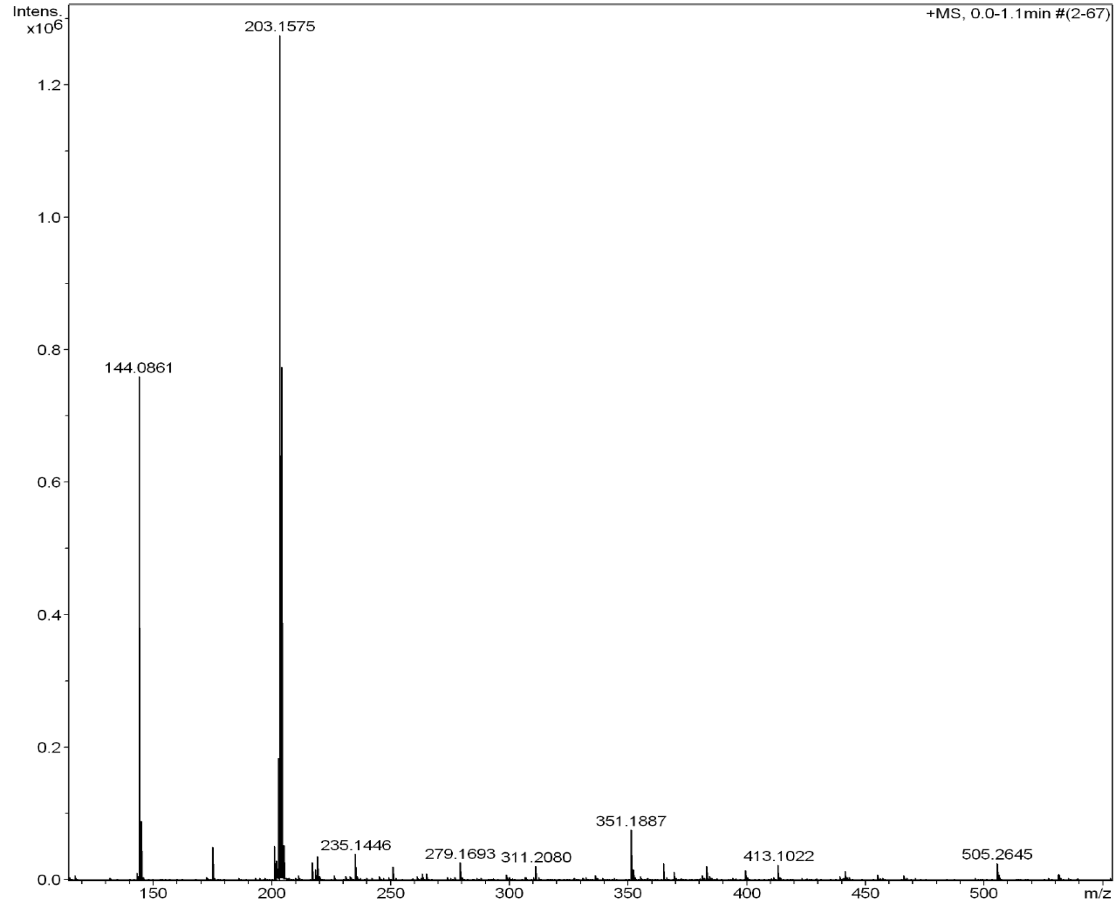

Figure S12. High Resolution Mass Spectrum (MS1) of compound **11**.

Analysis Info

Analysis Name	D:\Data\Ivo Cursino\MPN29 MSMS.d	Acquisition Date	9/10/2015 11:29:09
Method	tune_low_pos_LC_Chris_11_06_2014.m	Operator	UENF
Sample Name	MPN29 MSMS	Instrument	micrOTOF-Q II 10374
Comment			

Acquisition Parameter

Source Type	ESI	Ion Polarity	Positive	Set Nebulizer	2.0 Bar
Focus	Not active	Set Capillary	4500 V	Set Dry Heater	200 °C
Scan Begin	50 m/z	Set End Plate Offset	-500 V	Set Dry Gas	8.0 l/min
Scan End	1200 m/z	Set Collision Cell RF	120.0 Vpp	Set Divert Valve	Waste

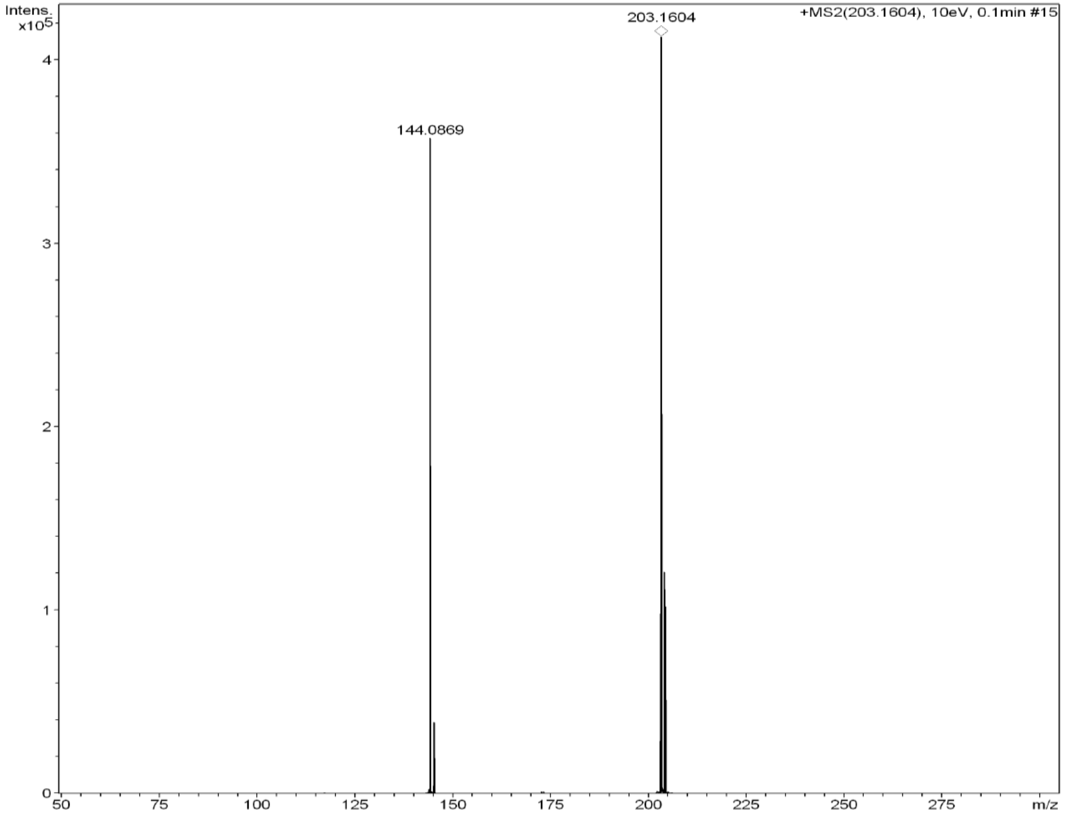

Figure S13. High Resolution Mass Spectrum (MS2) of compound **11**.

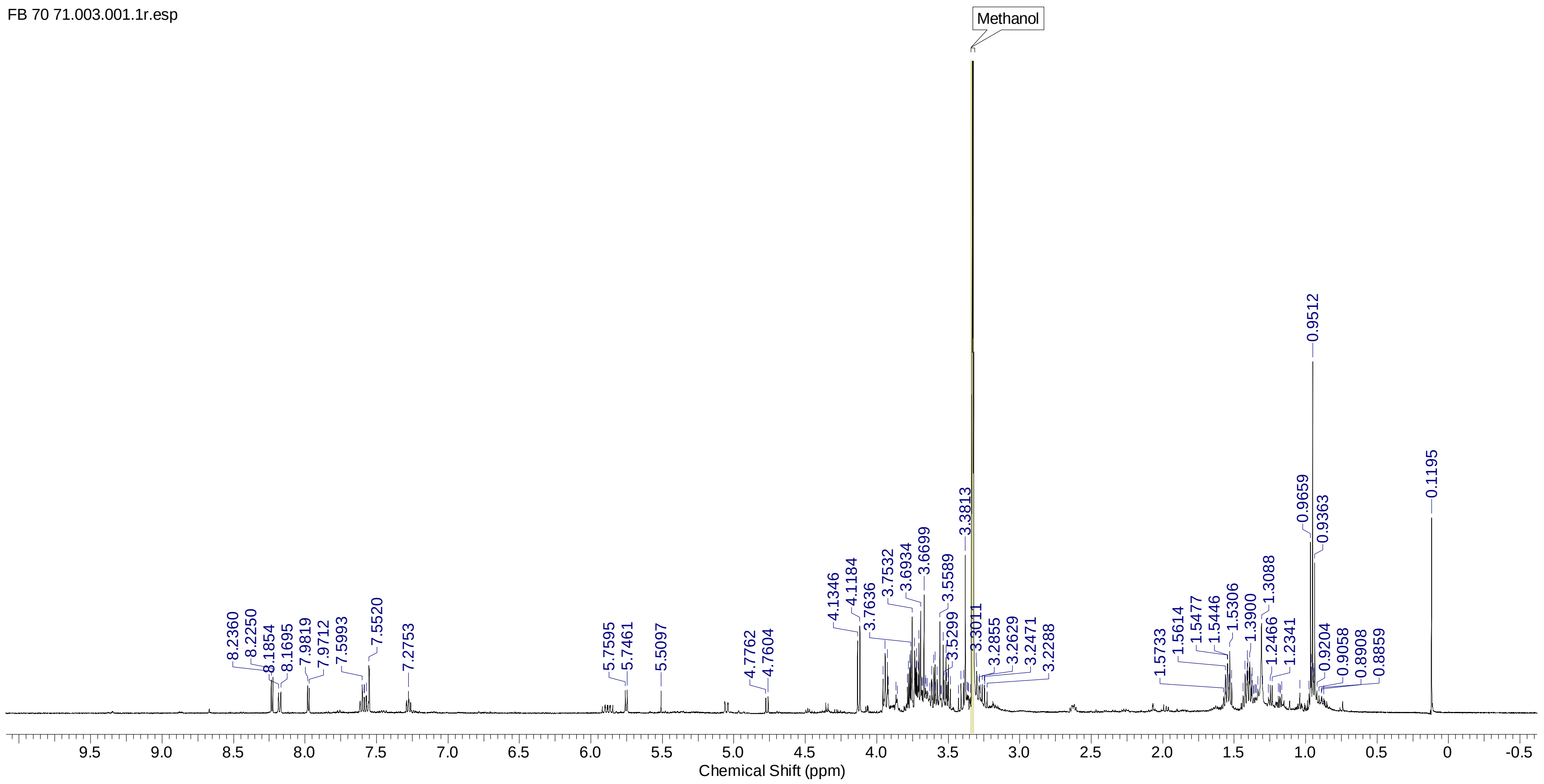

Figure S14. ^{1}H NMR spectrum (500 MHz, CD_3OD) of compounds **12** and **13**.

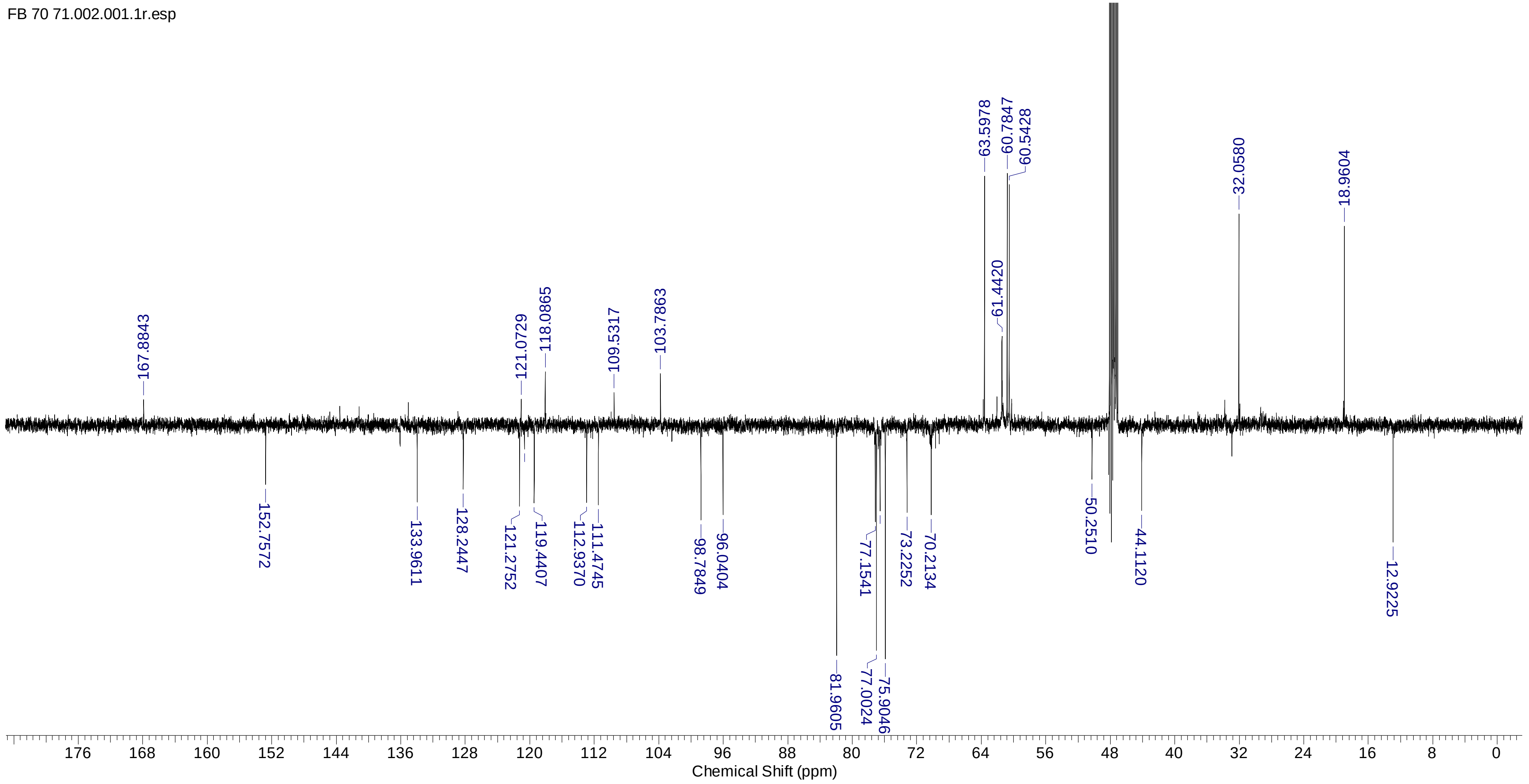

Figure S15. ^{13}C NMR spectrum (125 MHz, CD_3OD) of compounds **12** and **13**.

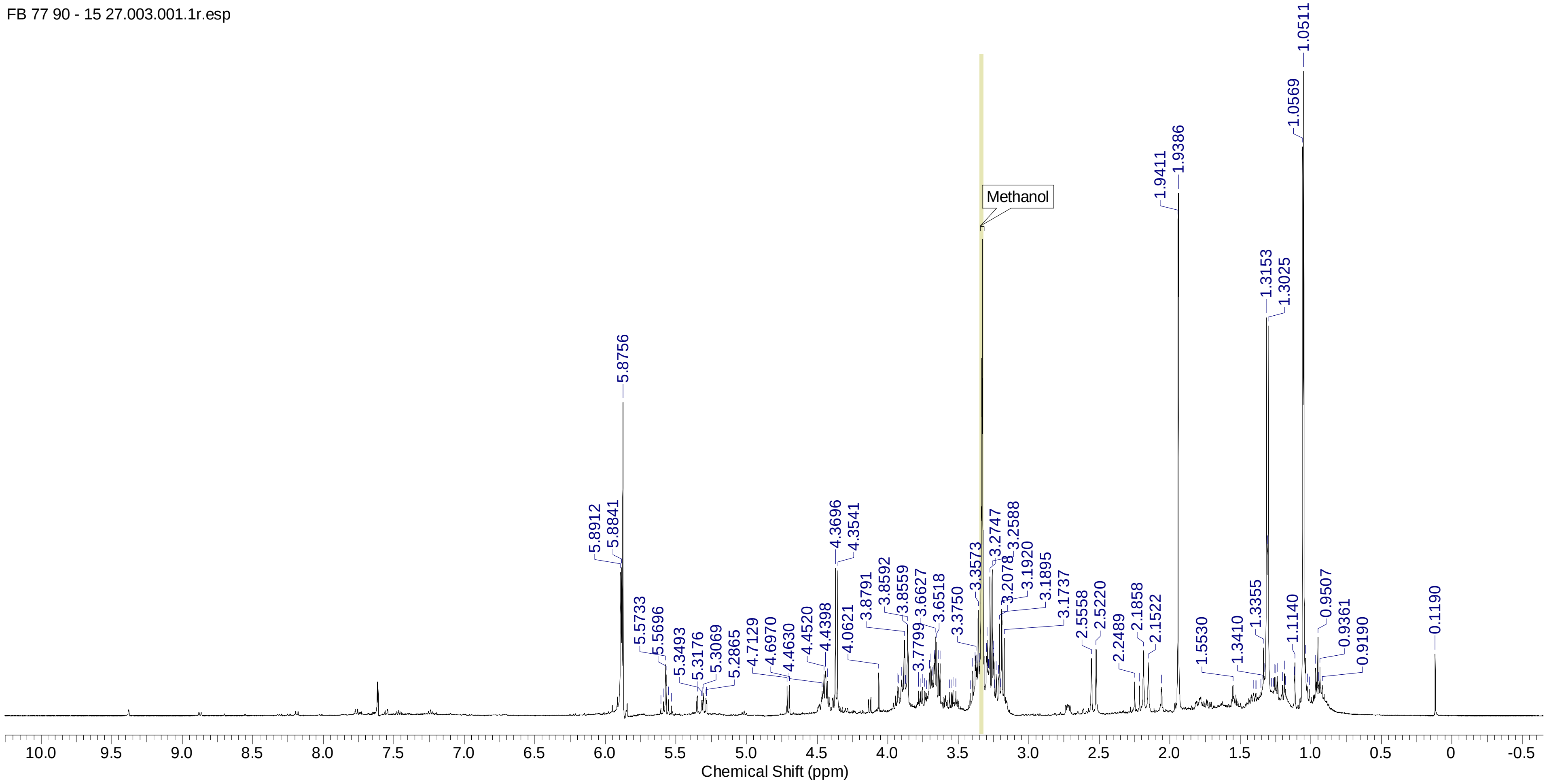

Figure S16. ^{1}H NMR spectrum (500 MHz, CD_3OD) of compound **14**.

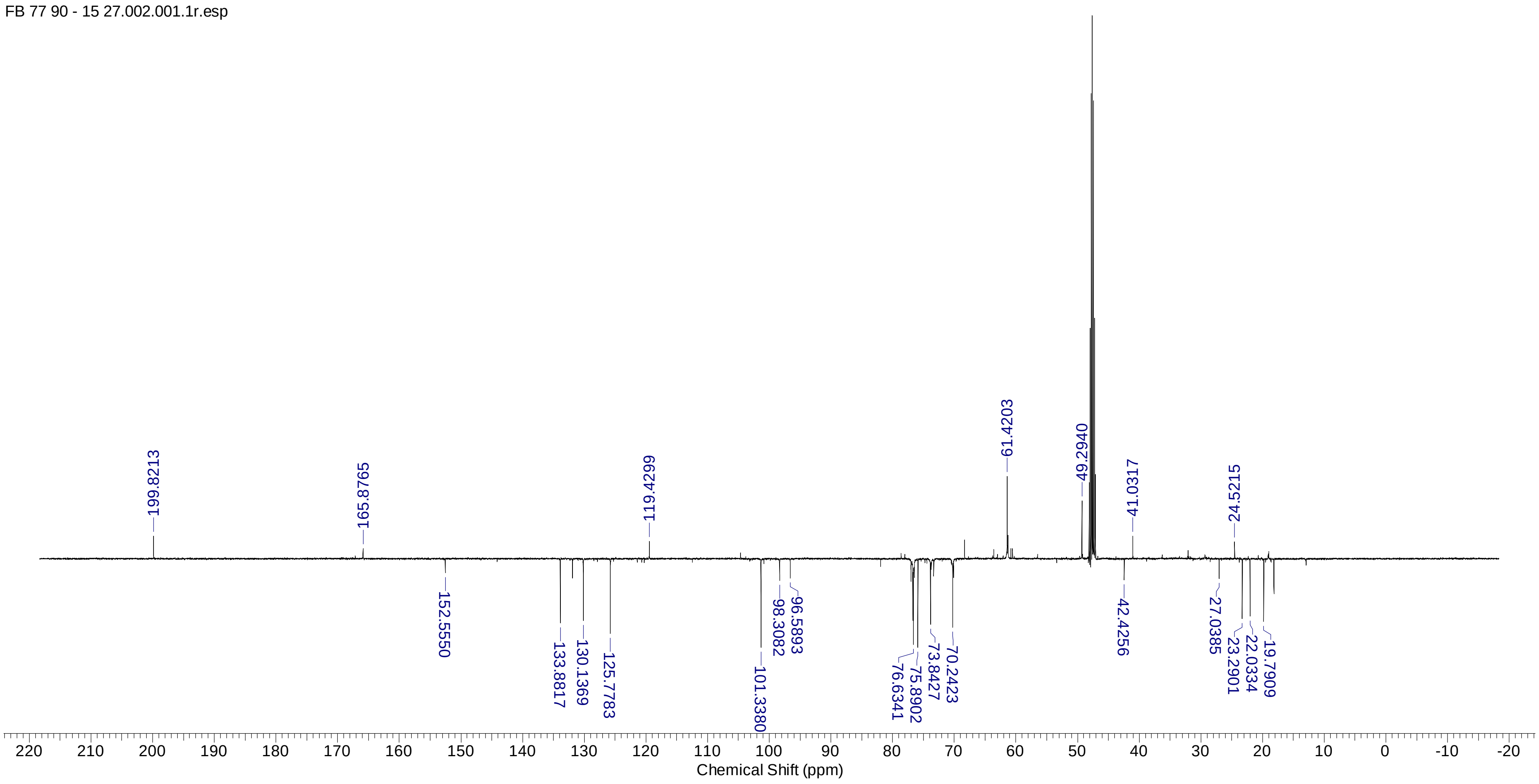

Figure S17. ^{13}C NMR spectrum (125 MHz, CD_3OD) of compound **14**.

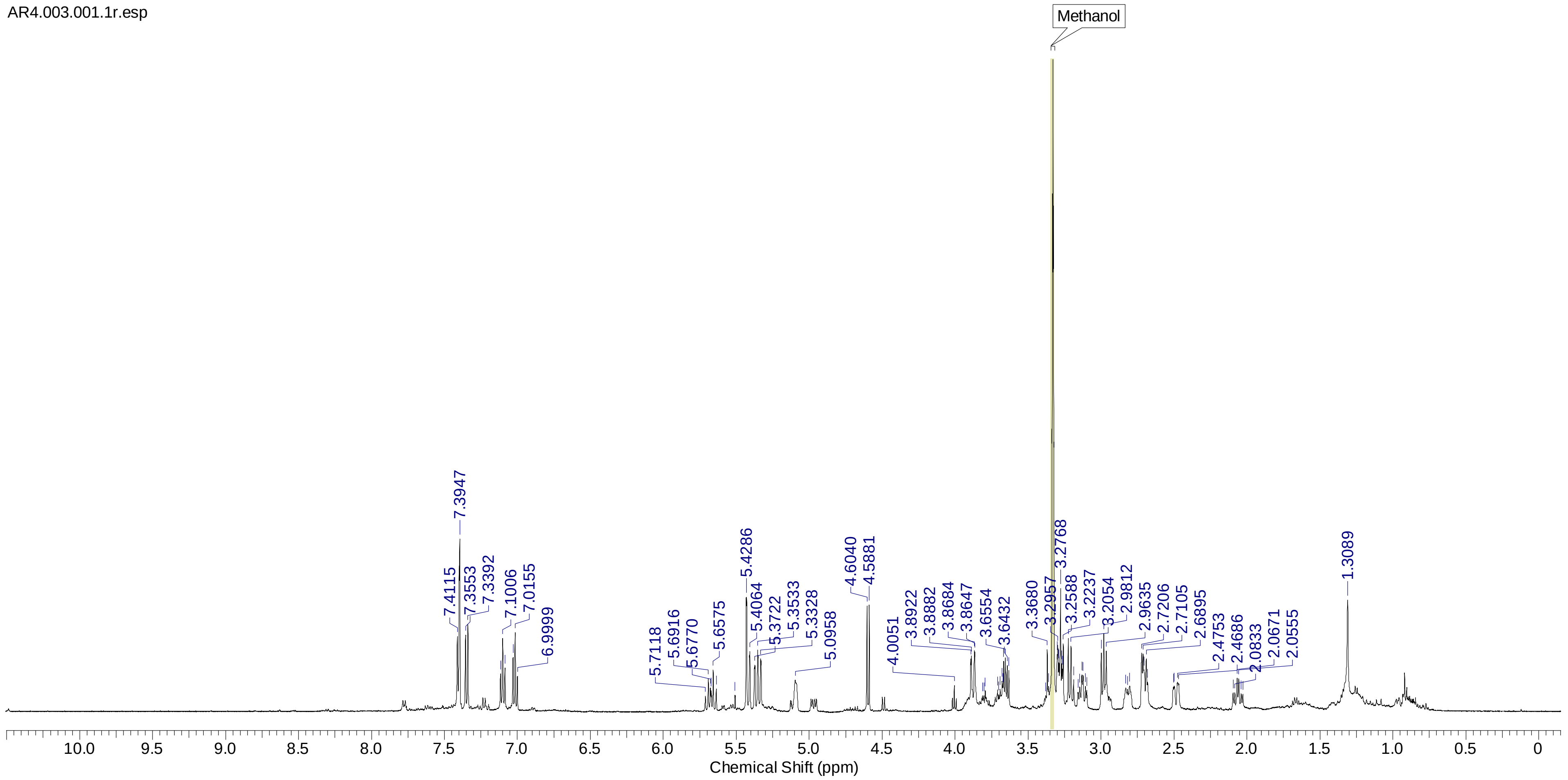

Figure S18. ^{1}H NMR spectrum (500 MHz, CD_3OD) of compound **15**.

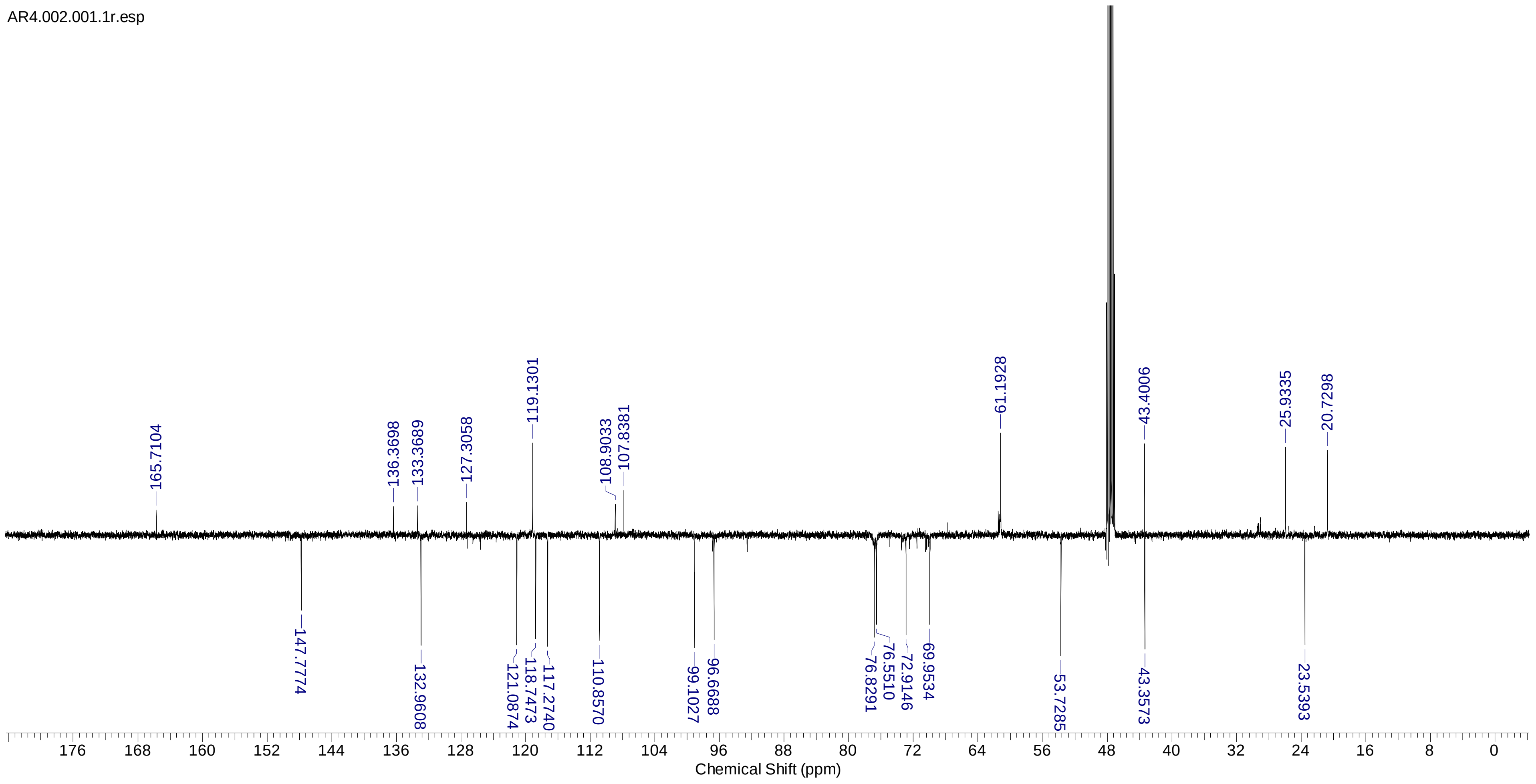

Figure S19. ^{13}C NMR spectrum (125 MHz, CD_3OD) of compound **15**.

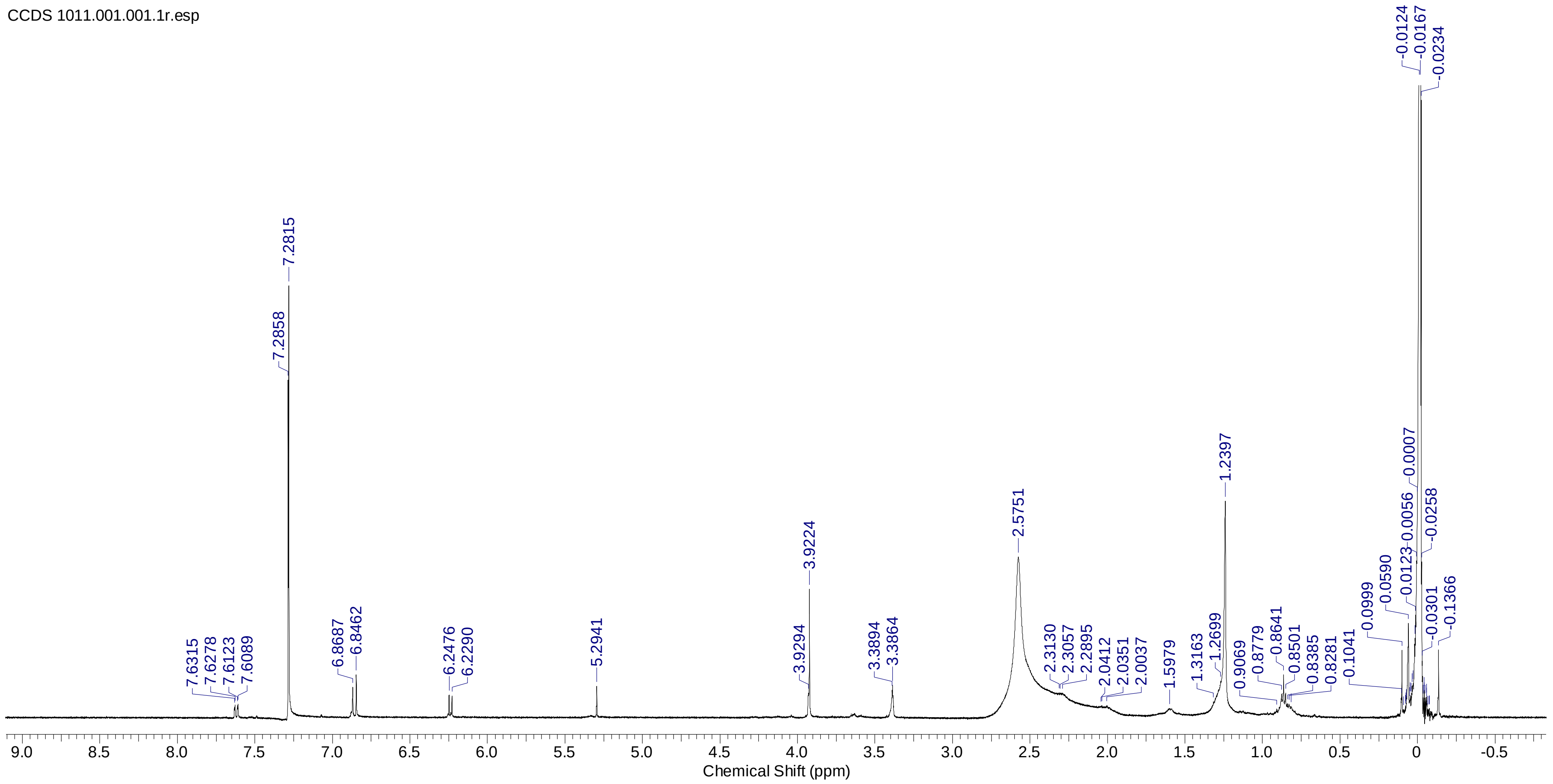

Figure S20. ^{1}H NMR spectrum (500 MHz, $CDCl_3$) of compound **16**.

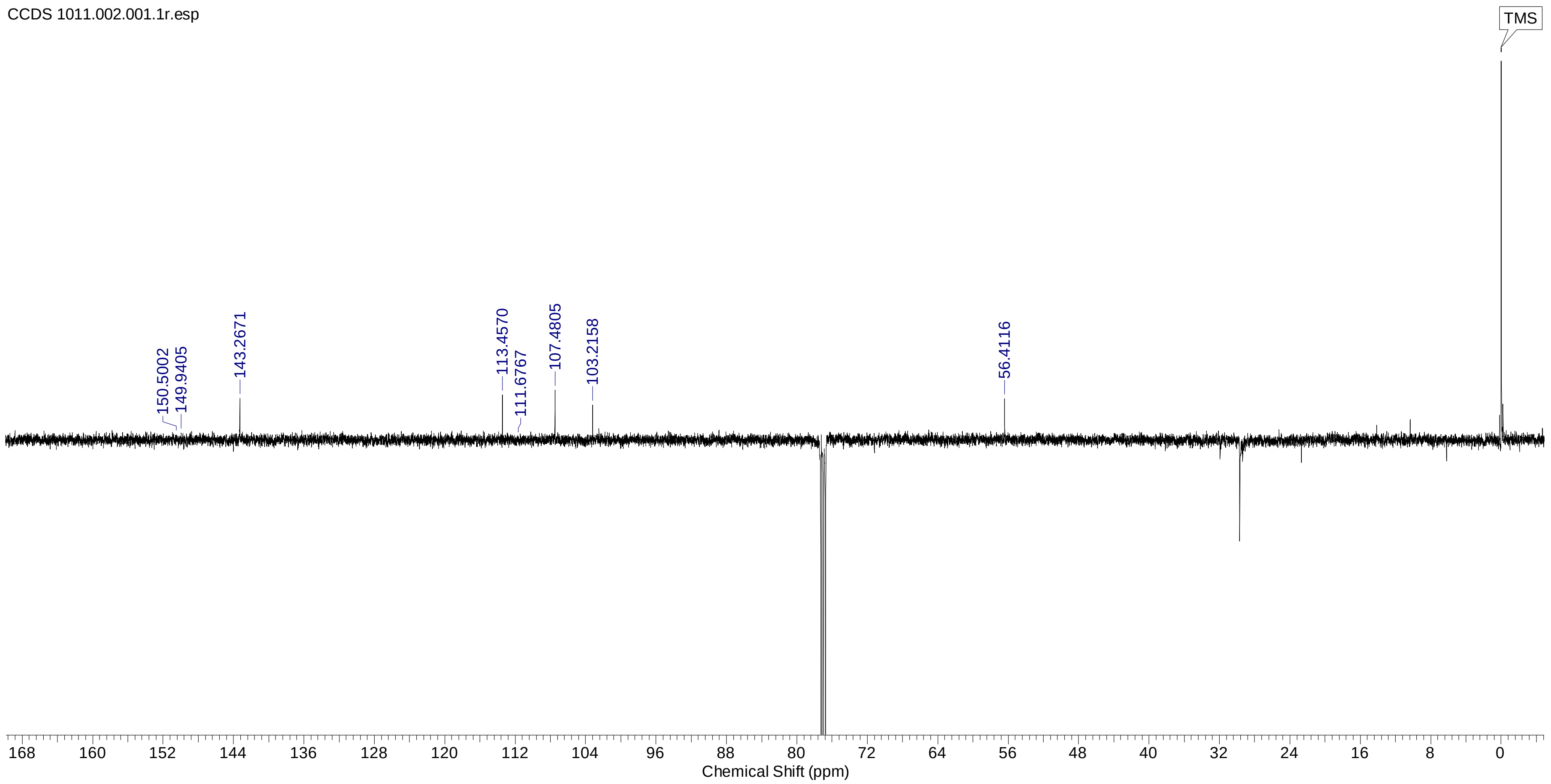

Figure S21. ^{1}H NMR spectrum (125 MHz, $CDCl_3$) of compound **16**.

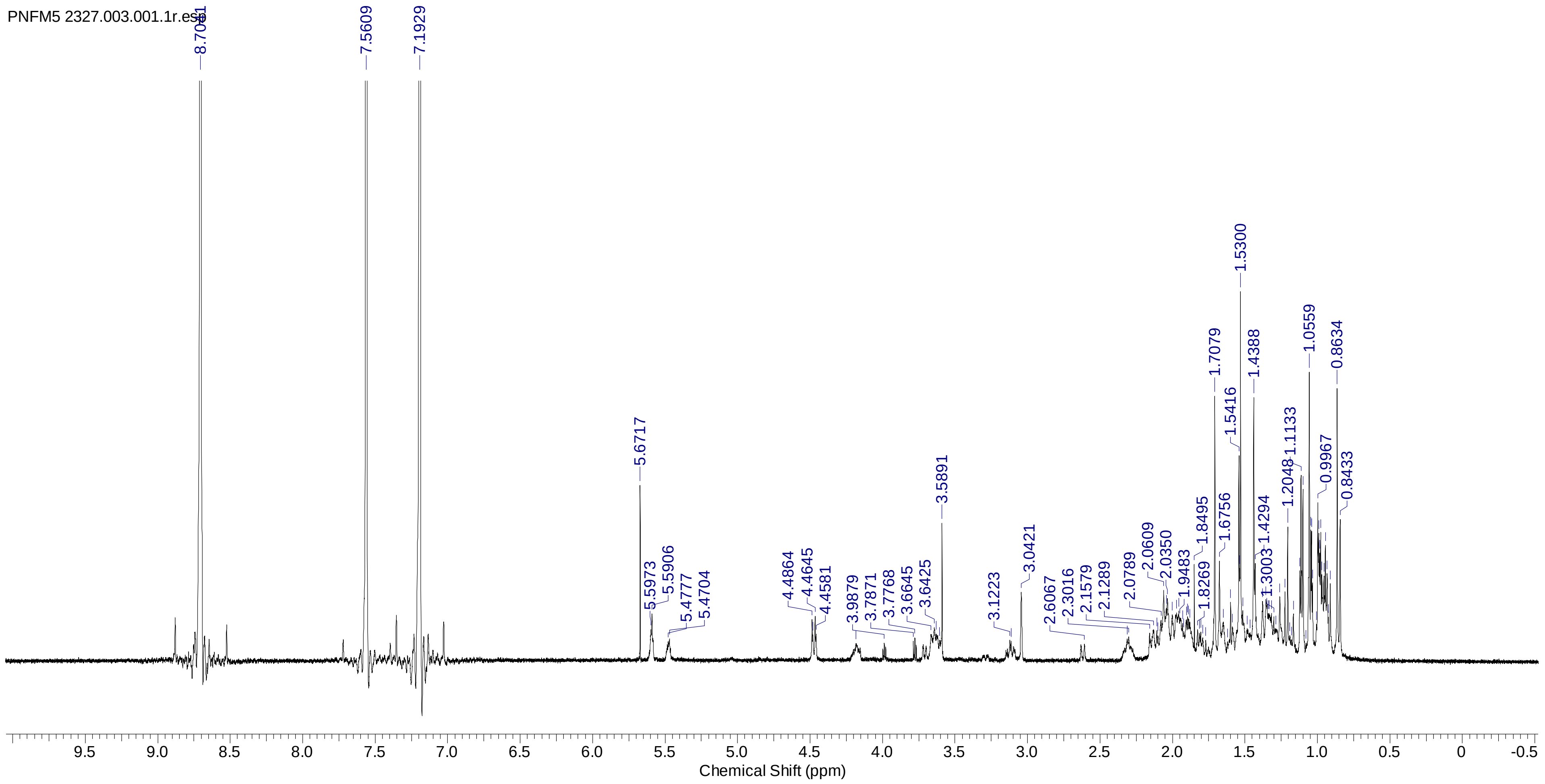

Figure S22. ^{1}H NMR spectrum (500 MHz, Pyridine-d5) of compound **17**.

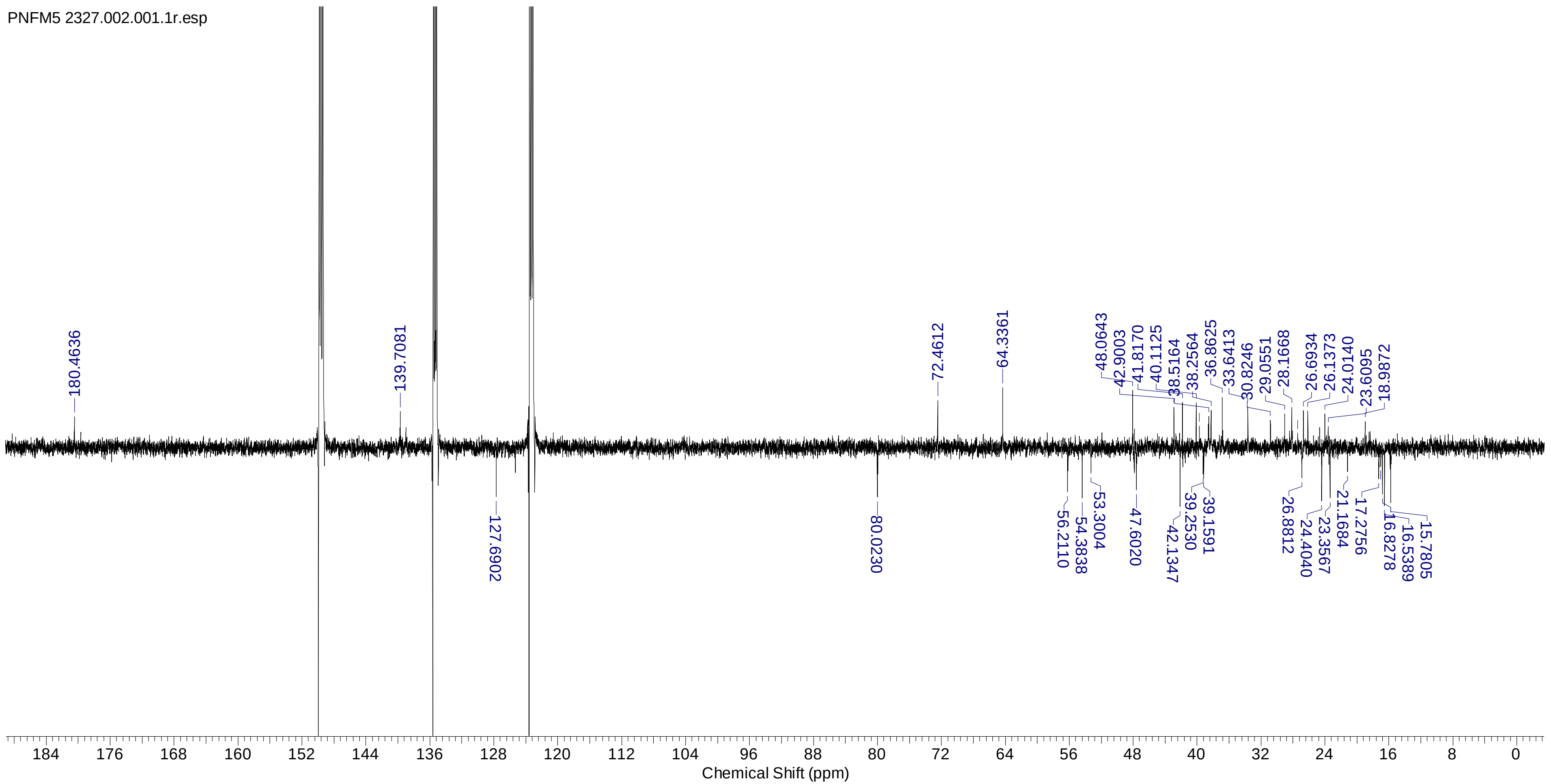

Figure S23. ^{13}C NMR spectrum (125 MHz, Pyridine-d_5) of compound **17**.

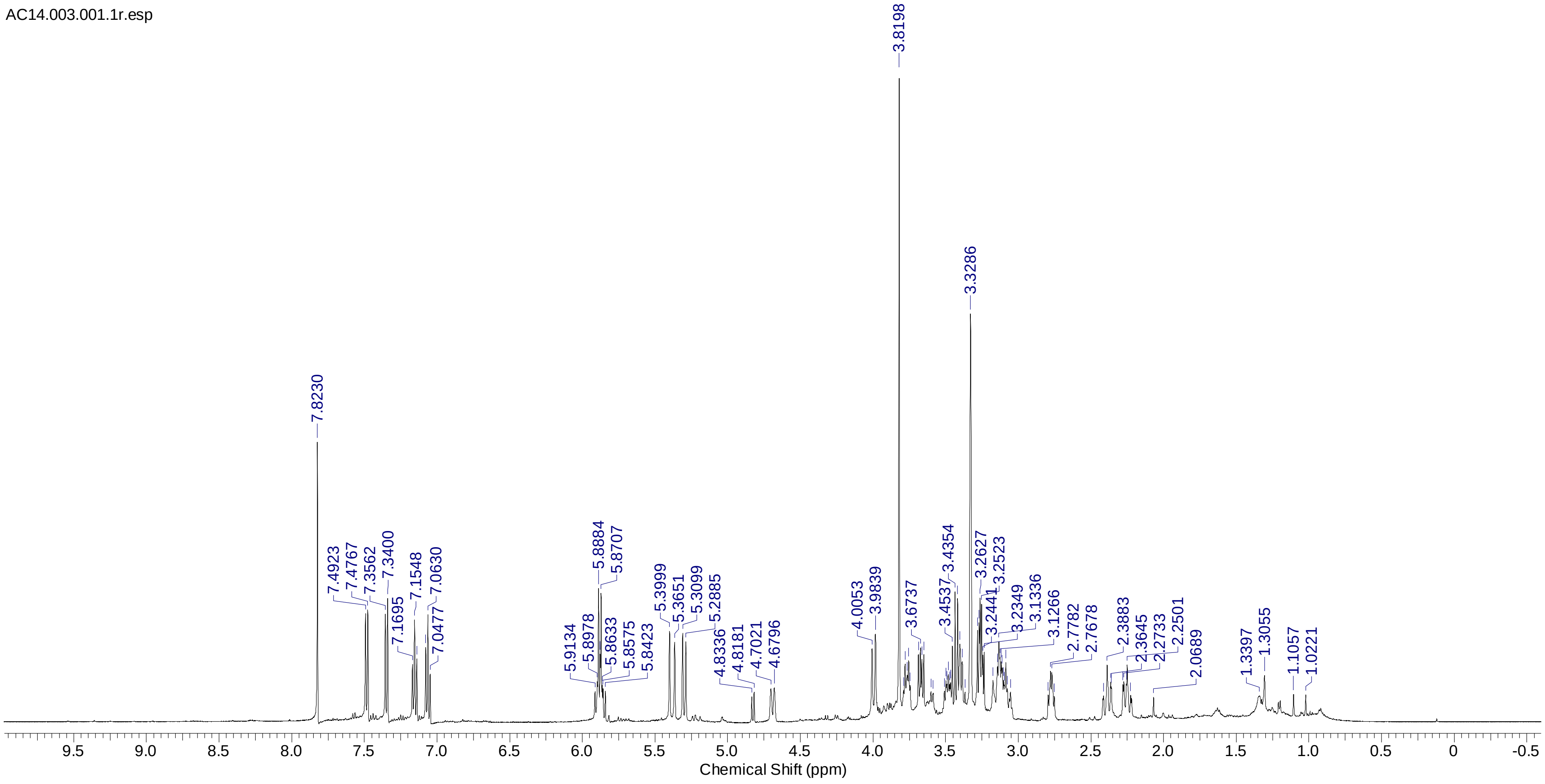

Figure S24. ^{1}H NMR spectrum (500 MHz, CD_3OD) of compound **18**.

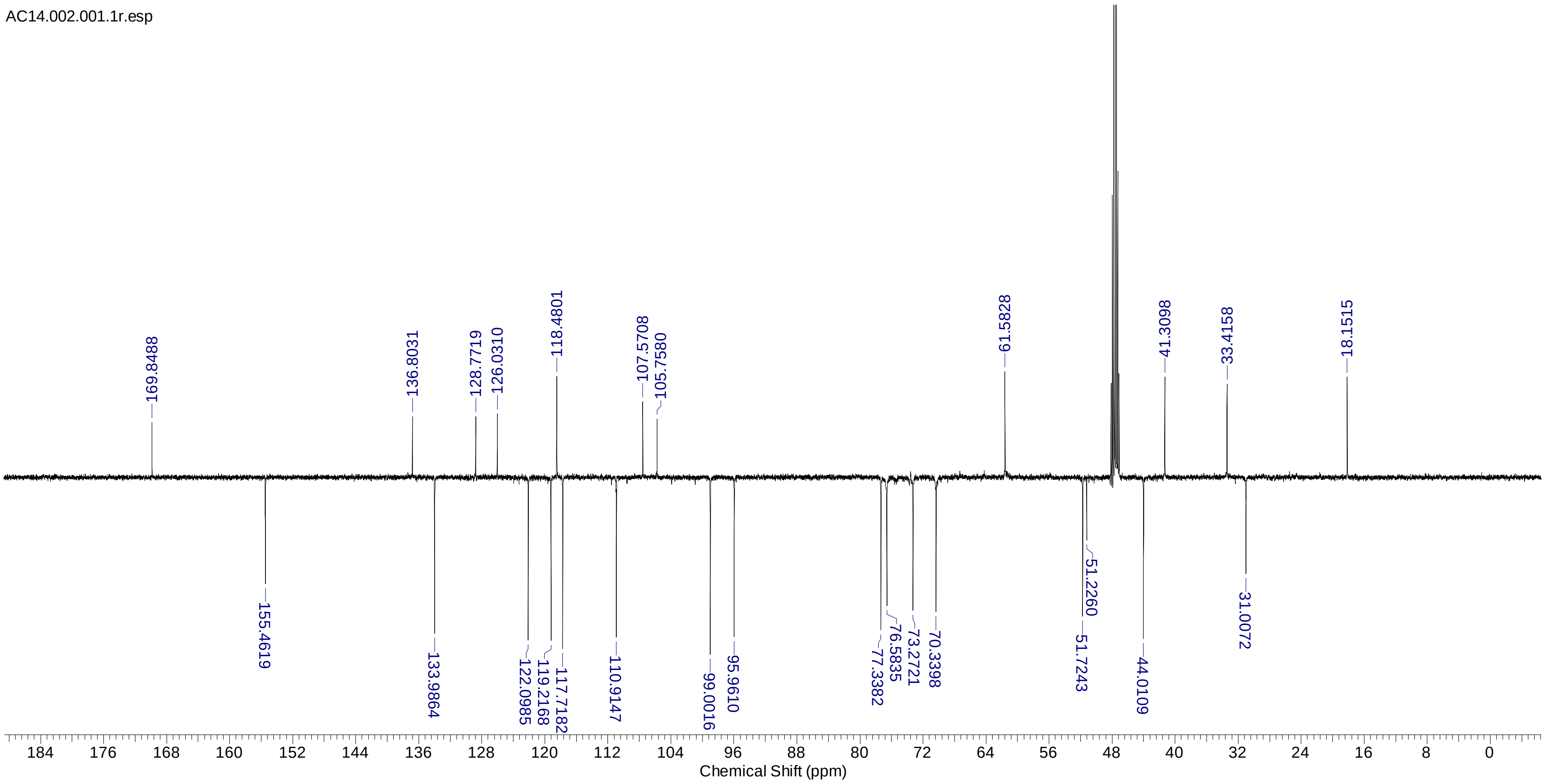

Figure S25. ^{13}C NMR spectrum (125 MHz, CD_3OD) of compound **18**.

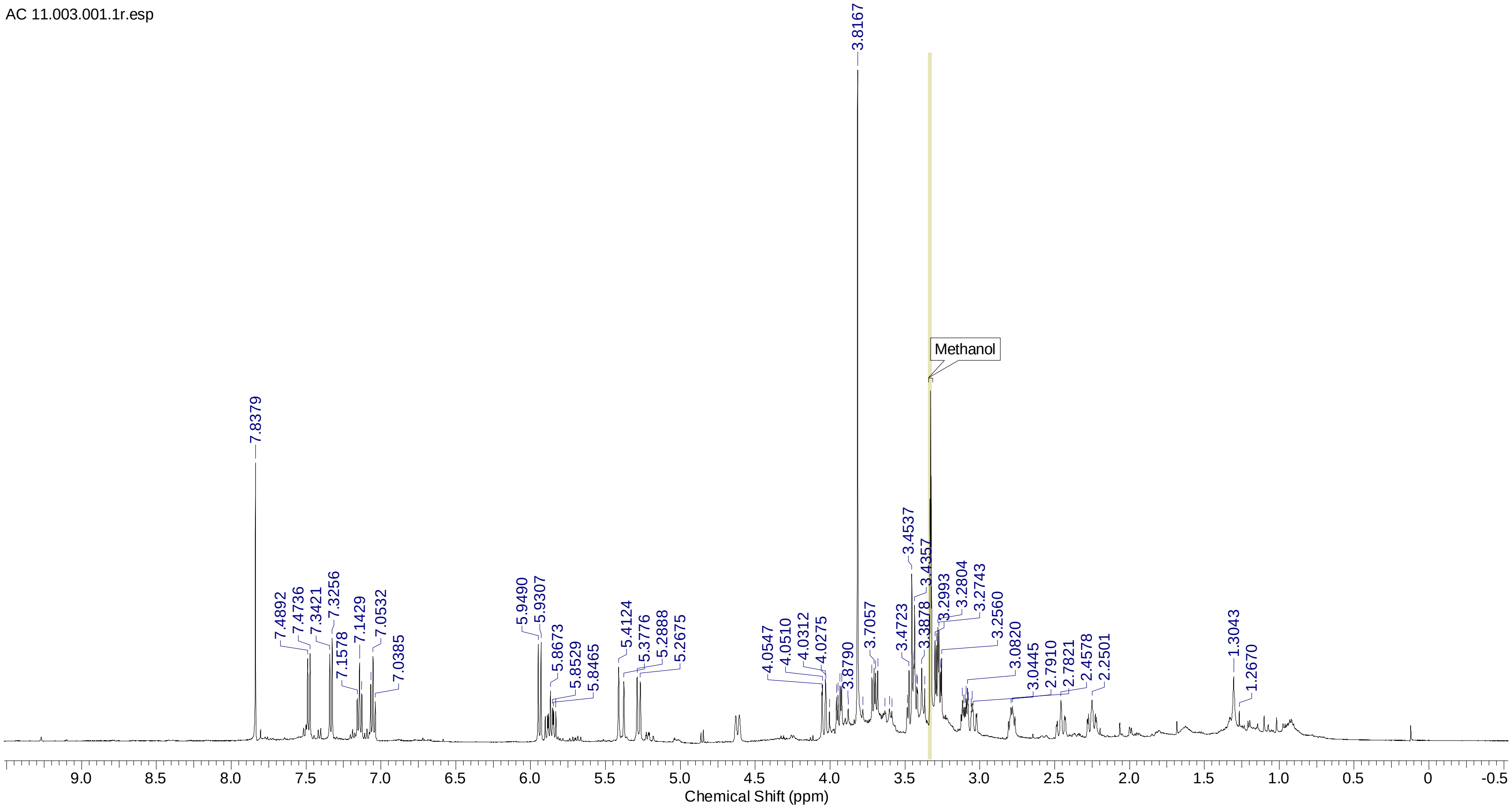

Figure S26. ^{1}H NMR spectrum (500 MHz, CD_3OD) of compound **19**.

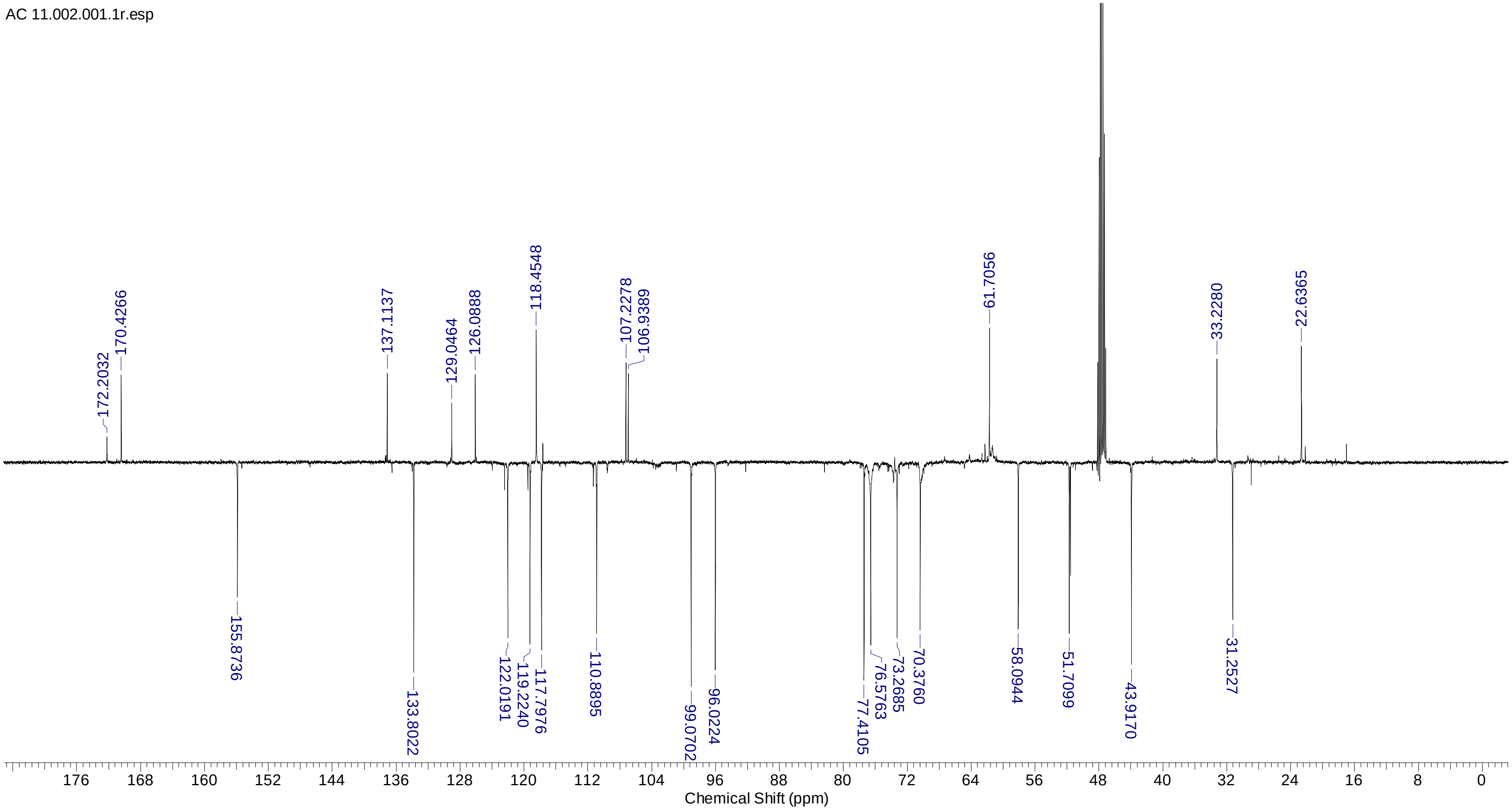

Figure S27. ^{13}C NMR spectrum (125 MHz, CD_3OD) of compound **19**.
